# Spontaneous Rupture of a Superior Gluteal Artery Mycotic Aneurysm

**DOI:** 10.14740/cr414w

**Published:** 2015-10-25

**Authors:** Neal George, Mahmoud Abdelghany, Owen Stark, Medha Joshi

**Affiliations:** a Department of Medicine, Conemaugh Memorial Medical Center, Johnstown, PA, USA; b Department of Interventional Radiology, Conemaugh Memorial Medical Center, Johnstown, PA, USA

**Keywords:** Superior gluteal artery, Mycotic aneurysm, Sciatica, Infective endocarditis, *Streptococcus viridans*

## Abstract

Gluteal artery aneurysms are uncommon among all aneurysms and are usually a result of trauma. *Streptococcus viridans* bacteremia has been described in rare cases of extracranial mycotic aneurysms. Despite a variable clinical presentation, mycotic aneurysms of the superior gluteal artery could be the cause in patients with unexplained sciatica pain. Here we report a very rare case of spontaneous rupture of a superior gluteal artery mycotic aneurysm in a patient with underlying infective endocarditis (IE) secondary to *Streptococcus viridans*.

## Introduction

In 1885, the term “mycotic aneurysm” was introduced to describe infected aneurysm secondary to embolism from bacterial endocarditis [1]. Embolization with clinical sequelae has been described in 13-44% of patients with infective endocarditis (IE); in most cases, embolization occurs prior to clinical presentation but can occur after initiation of antimicrobial therapy [2]. Systemic embolization most commonly occurs in left-sided IE, but superior gluteal artery is an exceptionally rare site for embolization. Aneurysmal rupture is a serious complication that might be life-threatening.

## Case Report

**Figure 1:**
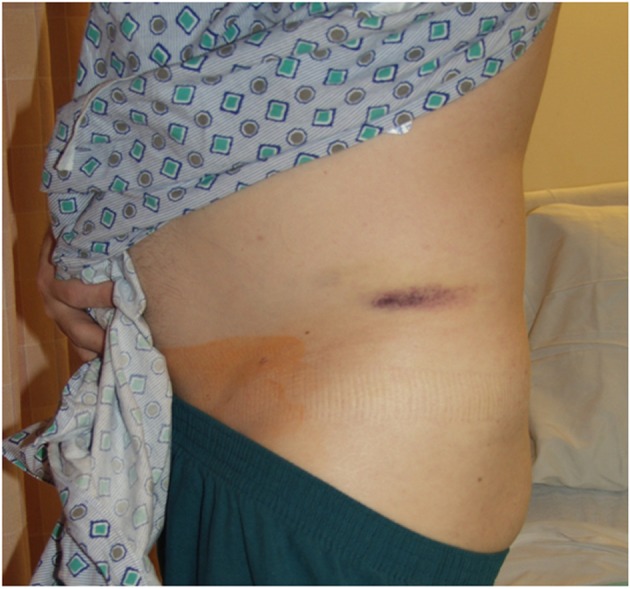
A photograph of the left side of the pelvis showing an area of ecchymosis over the left iliac crest.

**Figure 2:**
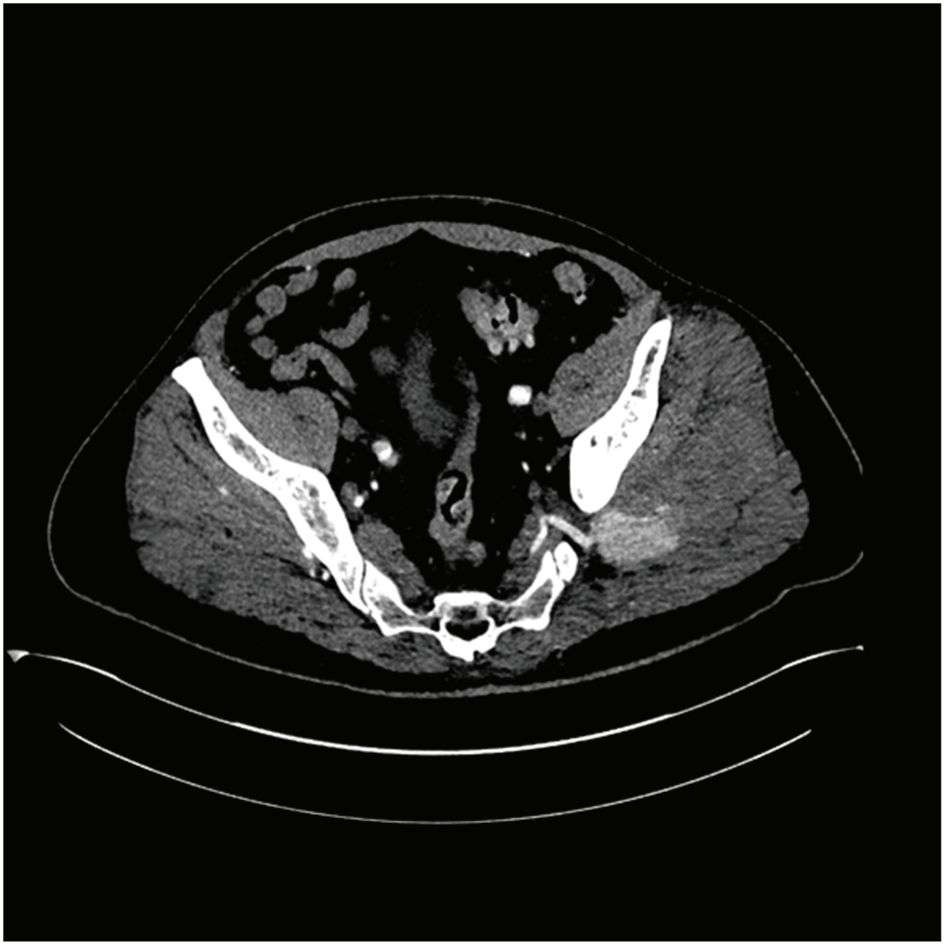
Contrast-enhanced computed tomography of the pelvis showing an area of contrast extravasations in the left gluteus medius muscle.

**Figure 3:**
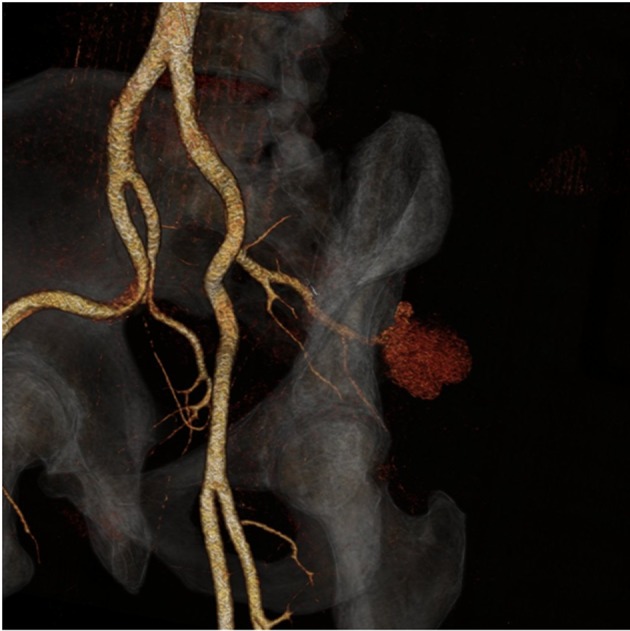
A 3D reformat of the contrast-enhanced computed tomography of the pelvis showing a ruptured aneurysm with an associated pseudoaneurysm.

**Figure 4:**
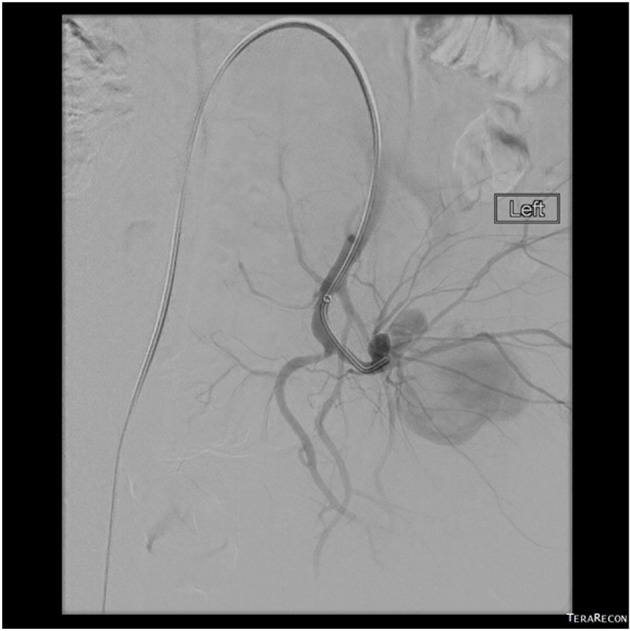
A selective left side internal iliac arteriogram showing a ruptured aneurysm with an associated large pseudoaneurysm.

A 49-year-old male, with no significant past medical history, presented to our hospital complaining of left buttock pain radiating to the left thigh. At the emergency department, he was clinically diagnosed with sciatica and was discharged home on naproxen. Two days later, the patient experienced sudden severe left buttock and flank pain after hearing a pop at this area with appearance of left flank hematoma (Fig. 1). On presentation, vital signs were as follows: temperature 38 °C, pulse 94/min, blood pressure 145/109 mm Hg, respiratory rate 18/min and oxygen saturations 95% on 2 L nasal cannula. The patient mentioned that he had an unintentional 30 pounds weight loss over 6 months. He complained of 10/10 left buttock pain. Blood work showed a hemoglobin of 10.3 g/dL and a hematocrit 32% (baseline is 15.7 g/dL and 45%, respectively), platelets 337,000 μL and sedimentation rate 61 mm/h. Contrast-enhanced computed tomography (CT) scan of the abdomen and pelvis (Fig. 2, 3), confirmed with an angiogram (Fig. 4), showed ruptured left superior gluteal artery aneurysm, with a 4.1 × 2.5 cm left gluteus medius muscle hematoma. The aneurysm was treated with coil embolization of the left superior gluteal artery using 5 mm × 5 cm MReye^®^ coil and 8 × 7 mm Amplatzer plug. ANA, C-ANCA, P-ANCA, RNP antibodies, and rheumatoid factor were negative. Two blood cultures from two different sites 1 h apart grew *Streptococcus viridians*. Empiric treatment with IV vancomycin was initiated and later adjusted, according to the cultures and sensitivity, to penicillin G. Transesophageal echocardiography showed aortic and mitral vegetations with anterior mitral leaflet perforation causing severe aortic and mitral regurgitation. The patient underwent aortic and mitral valve replacements and subsequently recovered without complications.

## Discussion

Aneurysms of the gluteal artery are very rare and represent 1% of all aneurysms. They predominantly originate from the superior rather than the inferior gluteal artery [3]. Most gluteal artery aneurysms result from trauma, pelvic fracture or iatrogenic causes. Mycotic aneurysms have been reported as a result of endocarditis in both pediatric and adult populations [4, 5]. In 1924, Benjamin and Lachman described the first reported case of mycotic gluteal artery aneurysm secondary to *Streptococcus viridans* [6]. A recent review of the literature revealed only six cases of mycotic aneurysm of the superior gluteal artery [7]. *Streptococcus viridans* has been reported in other rare cases of extracranial mycotic aneurysm including the superior mesenteric artery [8] as well as the popliteal artery [9]. The clinical presentations of these lesions are variable. Those include a pulsatile painful buttock mass, abdominal pain, nausea, vomiting, local hematoma, retroperitoneal hemorrhage and neurological deficit mainly due to compression of the sciatic nerve [3]. Compartment syndrome is a well-recognized complication, most commonly encountered in the lower leg, but could also occur in the gluteal region [10]. Doppler ultrasound could be used to confirm the arterial origin of the mass; however, CT or magnetic resonance imaging is more accurate for diagnosis. Angiography is a diagnostic and therapeutic modality [11].

The treatment of gluteal artery aneurysm has been, for a long time, exclusively through open surgery. Percutaneous embolization has been introduced as a safe and effective method to manage such aneurysms. Antibiotic therapy should be guided according to the blood and/or pathological specimen cultures. Vancomycin is a suitable option for empiric therapy. The duration for antibiotics remains debatable, and varies between 6 weeks and 6 months, and others suggest lifelong therapy [12].

### Conclusion

Gluteal artery aneurysms are exceedingly uncommon and should be considered in patients with unexplained buttock and sciatica pain. Patients with aneurysms or pseudoaneurysms at uncommon sites should have a complete diagnostic workup for IE in the right clinical setting.
